# Cultural adaptation and validation of an instrument on barriers for the use of research results^1^

**DOI:** 10.1590/1518-8345.1652.2852

**Published:** 2017-03-09

**Authors:** Maria Beatriz Guimarães Ferreira, Vanderlei José Haas, Rosana Aparecida Spadoti Dantas, Márcia Marques dos Santos Felix, Cristina Maria Galvão

**Affiliations:** 2 PhD, Professor, Universidade de Uberaba, Uberaba, MG, Brazil.; 3 PhD, Visiting Professor, Universidade Federal do Triângulo Mineiro, Uberaba, MG, Brazil.; 4 PhD, Associate Professor, Escola de Enfermagem de Ribeirão Preto, Universidade de São Paulo, WHO Collaborating Centre for Nursing Research Development, Ribeirão Preto, SP, Brazil.; 5 Doctoral student, Universidade Federal do Triângulo Mineiro, Uberaba, MG, Brazil. Scholarship holder from Coordenação de Aperfeiçoamento de Pessoal de Nível Superior (CAPES), Brazil.; 6 PhD, Full Professor, Escola de Enfermagem de Ribeirão Preto, Universidade de São Paulo, WHO Collaborating Centre for Nursing Research Development, Ribeirão Preto, SP, Brazil.

**Keywords:** Nursing, Research, Evidence-Based Nursing, Validation Studies, Factor Analysis, Statistical, Organizational Culture

## Abstract

**Objective:**

to culturally adapt The Barriers to Research Utilization Scale and to analyze the metric validity and reliability properties of its Brazilian Portuguese version.

**Method:**

methodological research conducted by means of the cultural adaptation process (translation and back-translation), face and content validity, construct validity (dimensionality and known groups) and reliability analysis (internal consistency and test-retest). The sample consisted of 335 nurses, of whom 43 participated in the retest phase.

**Results:**

the validity of the adapted version of the instrument was confirmed. The scale investigates the barriers for the use of the research results in clinical practice. Confirmatory factorial analysis demonstrated that the Brazilian Portuguese version of the instrument is adequately adjusted to the dimensional structure the scale authors originally proposed. Statistically significant differences were observed among the nurses holding a Master's or Doctoral degree, with characteristics favorable to Evidence-Based Practice, and working at an institution with an organizational cultural that targets this approach. The reliability showed a strong correlation (r ranging between 0.77 and 0.84, p<0.001) and the internal consistency was adequate (Cronbach's alpha ranging between 0.77 and 0.82).

**Conclusion:**

the Brazilian Portuguese version of The Barriers Scale was valid and reliable in the group studied.

## Introduction

The growing demand to improve the quality of health services implies nursing's search for actions to implement Evidence-Based Practice (EBP), aiming to promote the increased quality of nurses' care and professional growth. In addition, the traditionalist and ritualistic practice of the profession is currently inadmissible^1^.

EBP is a problem-solving approach to deliver health care that integrates the best evidence originating in well-designed studies and care data, in combination with the patient's preferences and values and the health professional's expertise^2^.

Hence, the implementation of EBP can offer benefits for the patient, health service and professionals working in the area, including the nurse. This approach increases the patient's access to information on effective treatments and can improve the institution, facilitating decision making consistently and at a lower cost. In addition, through information, it helps the nurse to make decisions, recycling these professionals by means of technologies and enhancing their efficiency^3^.

The use of research results in clinical practice is one of the components of EBP. Despite the increased volume of nursing research in many countries, transferring knowledge to practice remains a challenge. One of the actions that could minimize the gap between the knowledge produced and its application is the identification of barriers that impede the interdependence between research and practice^4^.

The Barriers to Research Utilization Scale was developed to investigate the barriers for the use of research results in clinical practice. The instrument consists of 29 items and three open questions. The items make up the four factors or domains of the scale. Factor 1 refers to the nurse's characteristics concerning the research, that is, value attributed, skills and knowledge, and includes eight items. Factor 2 pictures the characteristics of the organization where the research can be used, the barriers and limitations of this scenario, also including eight items. Factor 3 consists of six items, which reflect the research characteristics, such as methodological inadequacy and conflicting results in the literature. Factor 4 focuses on the communication characteristics (supply and accessibility of the research), consisting of six items, including the lack of readability and clarity of the study's implications for the practice^5^.

For each scale item, the respondent marks one out of five options on a Likert scale, in which scores 1 to 4 indicate the increase of the perceived barrier, and 5 that the participants does not opine. Thus, higher scores indicate greater barriers for the use of research results in practice. It is highlighted that item 27 in the scale is not scored, as it was not included in any of the factors. Nevertheless, the authors of the original instrument maintained this item based on the experts' assessments^5^.

The use of the scale can permit the identification of area lacking intervention, enhance the usage process of research results in practice, guide the development of educative programs, support dialogues among clinicians, researchers and administrators with a view to reducing gaps between research and its application^5^.

Studies exist in the literature in which the scale was validated for the context of the country^6^ ^-^ ^16^, as well as studies whose authors applied the instrument to identify the barriers for the implementation of evidence in the local practice^17^ ^-^ ^20^.

Due to the lack of research in the Brazilian literature on a measure to investigate the barriers for the use of research results in clinical practice, this study was intended to develop the cultural adaptation of the instrument The Barriers to Research Utilization Scale, as well as to analyze the metric properties of validity and reliability of its version for Brazilian Portuguese.

## Method

A methodological research was started after getting agreement from the main author of the instrument The Barriers to Research Utilization Scale.

The Barriers Scale was submitted to the cultural adaptation process proposed by experts on the procedure^21^, changing the order of the back-translation phase, which took place after the expert committee phase^22^. This change is justified, as it maintains the objective of the back-translation, which is to observe possible errors of meaning between the adapted version and the original version. That would not be the case if the adapted version were further modified by the expert committee^22^.

Hence, two independent persons (research and Brazilian teacher of English) translated the original version of the instrument into Brazilian Portuguese, resulting in a single version after the consensus meeting. The single version was submitted to an expert committee for assessment. The experts were selected based on their knowledge on EBP and their development of nursing research (four nurses/faculty holding a Ph.D.) and one professional mastering English. The experts assessed the cultural, semantic, conceptual and idiomatic equivalence, as well as the face and content validity. They requested that the original layout of the scale be mentioned and that modifications be made in the formulation of some instrument items (examples: from "a document on the need for change" to "a documented need to change the"; from "finds" to "has access to"). The changes the experts suggested resulted in the Brazilian Portuguese version of the instrument The Barriers Scale, which two independent translators translated to English: during a consensus meeting, the single version was formulated, which was then forwarded to the main author of the original version for evaluation, who answered that he had no contributions to add.

Confirmatory factor analysis was used to test the dimensional construct validity, and the known groups technique to test the construct validity, delimited by professional and academic criteria as well as by the workplace (hospital where EBP had been incorporated in the organizational culture and institution that did not adopt this approach in its organizational culture). Reliability was analyzed by means of internal consistency and the test-retest phases, with a seven-day interval, similar to the analysis developed in the original version of the instrument.

The study was developed at two hospitals, called A and B. Hospital A has not incorporated EBP into its organizational culture. It is located in the interior of the state of Minas Gerais and, at the time of the data collection, 184 nurses were serving on its staff. Hospital B does have this culture and is located in the city of São Paulo, with 542 nurses serving on the staff. The eligibility criteria of the participants were nurses working at the different hospital services with at least one month of formal employment at the sector.

For the sample size, the literature recommendations for confirmatory factor analysis were considered, that is, superior to at least 200 participants^23^ and appropriate balancing of the sample between both hospital. Hence, it was determined that at least 300 nurses should participate. All nurses at hospital A who complied with the criteria were invited to participate in the study. At hospital B, a draw was held but, due to the low adherence, we decided to invite everyone until reaching the pre-established figure. The study sample consisted of 335 nurses (hospital A=164; hospital B=171). For the retest phase, using a draw, 43 nurses answered the measure twice at distinct times.

The data were collected between October 2014 and June 2015, applying the instruments by means of a software model, which allowed the nurse to answer in logical order to complete the three delimited phases (Free and Informed Consent Term, sociodemographic and professional characteristics and The Barriers Scale-Brazilian Portuguese version). The application of the instruments was available on-line on the website http://www.thebarriers.com.br. By e-mail, the nurses received an explanation about the importance of the study and the access link. For the participants without e-mail, the instruments were answered in the presence of the researcher, at a pre-arranged time and place, at a reserved room without the influence of third parties.

In the data analysis, descriptive analyses were developed of the instrument items, identifying central trend and dispersion measures. The dimensional construct validity was assessed by means of the confirmatory factorial analysis, and the construct validity by means of known groups was investigated using Student's t-test, predicting higher scores for the group of nurses with characteristics unfavorable to EBP and working at the hospital with an organizational culture that does not target that approach. Cohen's d was also adopted to classify the magnitude of the difference between the mean scores of the groups or criteria studied as small (d<0.20), moderate (≥0.20 to <0.50) and big (≥0.50). The reliability was verified using the Intraclass Correlation Coefficient, with appropriate coefficients >0.70 and Pearson's Correlation Coefficient, considering the magnitude of the correlation as weak (0-0.29), moderate (0.30-0.49) and strong (≥0.50). The assessment of the internal consistency of the instrument items was calculated using Cronbach's alpha, acceptable coefficients being superior to 0.70. Significance was set at 0.05.

Approval for the research project was obtained from a Research Ethics Committee (protocol 844.856), according to Resolution 466/2012, and all participants signed the Free and Informed Consent Form.

## Results

Among the 335 participants, 297 (88.70%) were women. The average age was 33.2 years (minimum 23, maximum 69 years). The majority (272; 81.2%) held a *stricto sensu* post-graduation degree (Master's) and had no other employment bond (285; 85.1%). Table 1 displays the data on the research variables related to the training and use of research results in clinical practice.

**Table 1:** Distribution of the nurses according to professional characteristics, considering the variables related to the training and use of research results in clinical practice. Uberaba, MG, São Paulo, SP, Brazil, 2014, 2015

Variables		Hospital A			Hospital B			Total	
		n	%		n	%		n	%
Training about use of research results in practice									
	Yes	93	56.7		124	72.5		217	64.8
	No	71	43.3		47	27.5		118	35.2
Course on the use of research results in practice									
	Yes	21	12.8		29	17.0		50	14.9
	No	143	87.2		142	83.0		285	85.1
Training on search for scientific evidence in databases									
	Yes	48	29.3		114	66.7		162	48.4
	No	116	70.7		57	33.3		173	51.6
Reading of scientific articles on nursing practice									
	Yes	137	83.5		161	94.2		298	89.0
	No	27	16.5		10	5.8		37	11.0
Nursing research development									
	Yes	112	68.3		117	68.4		229	68.4
	No	52	31.7		54	31.6		106	31.6

The presence of professional characteristics favorable to the use of research results in practice was greater in the group of nurses from hospital B, highlighting the reading of scientific articles about nursing practice (161, 94.2%).

As regards the response frequencies for the items of The Barriers Scale - Brazilian Portuguese version, the nurses at hospital A identified the following as the main barriers: item 18-Physicians will not cooperate with implementation (50.6%), 6-The facilities are inadequate for implementation (40.2%) and 13-The nurse does not feel (s)he has enough authority to change patient care procedures (37.8%), all items belonging to Factor 2 (organizational characteristics). At hospital B, the main barriers the nurses reported also referred to Factor 2, that is: items 13 (35.7%); 18 (31%) and 29-There is insufficient time on the job to implement new ideas (24.6%). In the analysis of the average among the factors of the scale, Factor 2 also presented the highest mean score, and Factor 3 (research characteristics) the lowest.

**Figure 1:**
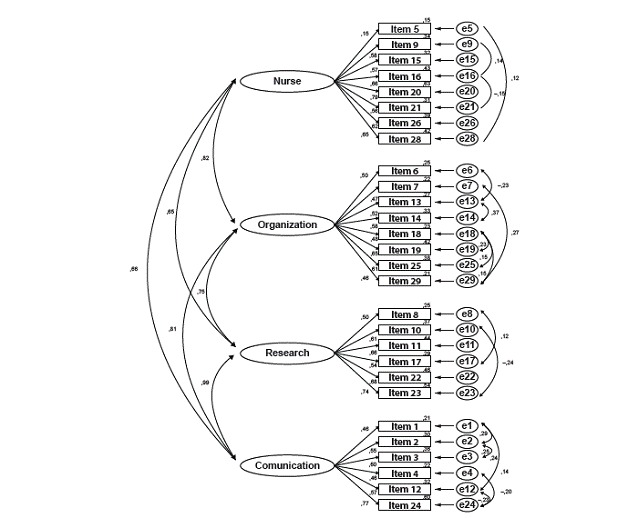
Diagram of confirmatory factor analysis of The Barriers Scale - Brazilian Portuguese version. Uberaba, MG, São Paulo, SP, Brazil, 2014-2015

In Figure 1, the results of the confirmatory factor analysis are presented to determine the dimensional construct validity of The Barriers Scale - Brazilian Portuguese version.

The model tested included a four-factor structure, containing the latent variables, indicated by the ellipses in Figure 1, characteristics of the nurse (Factor 1, containing eight items), characteristics of the organization (Factor 2, containing eight items), research characteristics (Factor 3, containing six items) and communication characteristics (Factor 4, containing six items). The items are indicated by rectangles and the factor loadings by the coefficients in the arrows.

As for the model fit indicators, concerning the absolute fit measures, the chi-square coefficient corresponded to χ2(327)=744.78, p<0.001, the root mean square error of approximation was RMSEA=0.062 (90% CI=0.056-0.068) and the goodness of fit index was GFI=0.87; while the Tuker-Lewis Index corresponded to TLI=0.86 and the Comparative Adjustment Index to CFI=0.87. It is highlighted that, although the hypothesis of equality between the variance-covariance matrices needs to be rejected based on the chi-squared coefficient (predicted by the model and obtained based on the data), the RMSEA lies within the limits considered as indicating adequate fitness of the model to the proposed factorial structure. In addition, the incremental measures are very close to the cut-off point recommended (0.90) for adequate goodness of fit. Hence, it is concluded that the model is fit to the dimensional structure proposed in the original version of the measure.

To understand the data presented in Table 2, lower means represent lesser barriers as appointed by the participants, while higher means indicate greater barriers. In descriptive terms, the means of all variables were lower when characteristics favorable to EBP were present, demonstrating that, when the nurse possesses these characteristics, in her opinion, she indicated lesser barriers to use the research results in practice.

**Table 2:** Mean (x̅), standard deviation (s) and effect size (Cohen's d) for the construct validity, considering the research variables, for each of the factors of The Barriers Scale. Uberaba, MG, São Paulo, SP, Brazil, 2014, 2015

		Factor 1				Factor 2					Factor 3					Factor 4			
		x̅	S	p	d		x̅	s	P	d		x̅	s	p	d		x̅	s	p	d
Institution																				
	Hospital A	2.48	0.66	0.25	0.14		2.97	0.54	<0.001	0.54		2.53	0.64	0.001	0.38		2.62	0.62	<0.001	0.54
	Hospital B	2.39	0.66				2.65	0.61				2.29	0.61				2.28	0.61		
Training on use of research results																				
	Yes	2.35	0.66	0.001	0.38		2.74	0.60	0.01	0.30		2.33	0.63	0.01	0.30		2.39	0.63	0.03	0.25
	No	2.56	0.64				2.92	0.59				2.53	0.64				2.55	0.63		
Course on use of research results																				
	Yes	2.42	0.65	0.84	0.03		2.73	0.58	0.36	0.15		2.24	0.64	0.05	0.30		2.24	0.66	0.01	0.40
	No	2.44	0.67				2.82	0.60				2.43	0.63				2.49	0.62		
Database search																				
	Yes	2.39	0.67	0.19	0.15		2.71	0.59	0.004	0.32		2.33	0.62	0.05	0.22		2.33	0.64	0.001	0.39
	No	2.49	0.66				2.90	0.59				2.47	0.65				2.57	0.60		
Reading articles																				
	Yes	2.41	0.66	0.02	0.39		2.78	0.60	0.02	0.39		2.37	0.63	0.008	0.49		2.41	0.63	0.005	0.52
	No	2.67	0.66				3.01	0.54				2.68	0.68				2.73	0.55		
Research development																				
	Yes	2.41	0.66	0.23	0.14		2.80	0.59	0.84	0.03		2.36	0.61	0.12	0.20		2.39	0.63	0.01	0.30
	No	2.50	0.67				2.82	0.62				2.49	0.69				2.58	0.61		
Having another job																				
	Yes	2.49	0.59	0.58	0.09		2.96	0.53	0.05	0.30		2.51	0.54	0.19	0.21		2.59	0.52	0.08	0.27
	No	2.43	0.68		2.78	0.61		2.38	0.65		2.42	0.65

The nurses appointed lesser barriers when characteristics favorable to the use of research results in clinical practice were present, with statistically significant differences when they worked at an institution with an organizational culture targeting EBP for Factors 2, 3 and 4. The same was true when they received training on the use of research results offered by the institution, for all Factors; courses on the application of research for Factors 3 and 4; search for scientific evidence in databases for Factors 2, 3 and 4; research development for Factor 4 and single employment bond for Factor 2 (Table 2).

In descriptive terms, nurses holding a specialization degree experts identified greater barriers for all factors (Factor 1: x̅=2.54; Factor 2: x̅=2.88; Factor 3: x̅=2.52; Factor 4: x̅=2.66) when compared to the nurses holding a Master's or Doctoral degree (Factor 1: x̅=2.43; Factor 2: x̅=2.80; Factor 3: x̅=2.40; Factor 4: x̅=2.44), who identified lesser barriers. This difference was not statistically significant though, which made discrimination power impossible for the qualification variable, which distinguished the participants' education in the *lato sensu* and *stricto sensu* modalities.

In Table 2, Cohen's d is displayed, defined by the difference of means divided by the standard deviation of the difference, which indicates the effect size. Thus, it is considered that, the greater the effect, the greater the impact of the presence of a characteristic favorable to the nurse's practice based on EBP. For most variables, the effect size was moderate and high, except for Factor 1, concerning the institutional variable, search for scientific evidences in databases and having another job, as well as for Factors 1 and 2 concerning the course on the use of research results.

Forty-three nurses participated in the retest phase through a draw. Pearson's Correlation and the Intraclass Correlation Coefficient were used to assess the reliability. The correlations indicated similarity between the test-retest items. The Intraclass Correlation and Pearson's Correlation Coefficients evidenced excellent and strong reliability indicators, with a statistically significant difference for all factors. To assess the internal consistency of the items, Cronbach's alpha coefficients were calculated, which were acceptable: 0.92 (Barriers total), 0.82 (Factor 1), 0.78 (Factor 2), 0.78 (Factor 3) and 0.77 (Factor 4) (Table 3).

**Table 3:** Test-retest reliability analysis of The Barriers Scale, considering the factors. Uberaba, MG, São Paulo, SP, Brazil, 2014-2015

	Test		Retest			ICC*	P		r**^†^**	p
	x̅	s	x̅	S				
Factor 1	2.47	0.58	2.46	0.60	0.84	<0.001	0.84	<0.001
Factor 2	2.97	0.48	3.06	0.48	0.83	<0.001	0.83	<0.001
Factor 3	2.51	0.55	2.45	0.59	0.82	<0.001	0.82	<0.001
Factor 4	2.69	0.54	2.65	0.44	0.75	<0.001	0.77	<0.001

*Intraclass Correlation Coefficient

†Pearson Correlation Coefficient

## Discussion

The methodological research results on The Barriers Scale were similar to this study concerning the female sex and the age^5^ ^,^ ^7^ ^,^ ^11^ ^-^ ^16^. The habit of reading scientific articles and research development characterized most of the nurses in this study. Differently from a study involving Spanish nurses, whose results evidenced that the professionals possessed less than 40 hours of non-formal preparation for research (455; 69.15%), and had done their most recent scientific reading between the past month and more than one year earlier^15^.

Based on the analysis of the methodological studies identified in the literature, it can be affirmed that the main barriers the nurses reported were similar to the barriers reported in this study, in that they belonged to Factor 2 (organizational characteristics), more specifically items 6 and 13^6^ ^-^ ^8^ ^,^ ^10^ ^-^ ^13^ ^,^ ^15^.

In a study developed in Turkey, involving 300 nurses from four hospitals, the results indicated the goodness of fit of the proposed factorial model, similar to this study, demonstrating that the Turkish version of the scale consisted of the same four factors as the original version of the measure. The factor loadings varied between 0.58 and 0.84 for Factor 1; 0.56 and 0.95 for Factor 2; 0.69 and 0.88 for Factor 3 and between 0.48 and 0.97 for Factor 4. The indicators presented the goodness of fit of the model (GFI=0.97; RMSEA=0.06), and Cronbach's alpha for the general scale corresponded to 0.92, ranging between 0.73 and 0.80 for the factors^14^.

It is highlighted that, in the methodological studies, in which the authors developed exploratory factor analysis, the results evidenced factorial structures that differed from the original model^7^ ^-^ ^9^ ^,^ ^11^ ^,^ ^13^.

As for the discrimination of the scale's validity, it is highlighted that no methodological research has been identified in the literature that was developed in two realities (institution with organizational culture targeting EBP and another without that culture). In one study only, in the metric analysis of the Chinese version of The Barriers Scale*,* the authors used the qualification variable to estimate the construct validity for known groups, affirming that this validity rests on the fact that higher scores were observed among nurses with a higher education level. Nurses holding a Master's degree or higher presented higher mean barrier scores for the use of research results in (x̅=2.91) when compared to nurses holding an undergraduate degree (x̅=2.69; p=0.001)^16^.

These research results were different. Controversies exist in the literature. Nurses with higher education levels are expected to find it easier to apply the research results in clinical practice. Nevertheless, there is ongoing discussion on whether these professionals identify greater or lesser barriers for the use of research results, as it depends on the context they are working in. To give an example, Master's and Doctoral graduates working at an institution without an organizational culture that targets EBP tend to identify greater barriers to apply the research results in practice. On the other hand, if they work at a service with such organizational culture, they tend to identify lesser barriers. In view of the above, future studies are needed to analyze the relation between the identification of barriers for the implementation of research results in clinical practice and the organizational context of the health service.

No studies were identified in the literature whose authors assessed the reliability of the instrument using the test-retest phases, as executed in this study and in the original version of the scale^5^. In this research, the reliability of the instrument was assessed, using the analysis of the Intraclass Correlation Coefficient and of Pearson's Correlation Coefficient. The coefficients of the latter (between 0.77 and 0.84) were similar to the original study (between 0.68 and 0.83)^5^, indicating the temporal stability of the scale between two assessments.

## Conclusions

The Barriers Scale - Brazilian Portuguese version is a valid and reliable tool that is easy to apply and can be used at health services.

The assessment of its use in practice depends on the development of new studies in different contexts. Its application permits diagnosing the main barriers for nurses to use research results. The scale is a management tool that can further the understanding of the needs to promote the implementation of EBP, improving the quality of care, reducing health institutions' operating costs and benefiting nurses' evidence-based decision making process.
